# A Complex Chained P System Based on Evolutionary Mechanism for Image Segmentation

**DOI:** 10.1155/2020/6524919

**Published:** 2020-08-07

**Authors:** Xiyu Liu, Lin Wang, Jianhua Qu, Ning Wang

**Affiliations:** ^1^Institute of Management Science, Shandong Normal University, Jinan 250000, China; ^2^College of Business, Shandong Normal University, Jinan, China

## Abstract

A new clustering membrane system using a complex chained P system (CCP) based on evolutionary mechanism is designed, developed, implemented, and tested. The purpose of CCP is to solve clustering problems. In CCP, two kinds of evolution rules in different chained membranes are used to enhance the global search ability. The first kind of evolution rules using traditional and modified particle swarm optimization (PSO) clustering techniques are used to evolve the objects. Another based on differential evolution (DE) is introduced to further improve the global search ability. The communication rules are adopted to accelerate the convergence and avoid prematurity. Under the control of evolution-communication mechanism, the CCP can effectively search for the optimal partitioning and improve the clustering performance with the help of the distributed parallel computing model. This proposed CCP is compared with four existing PSO clustering approaches on eight real-life datasets to verify the validity. The computational results on tested images also clearly show the effectiveness of CCP in solving image segmentation problems.

## 1. Introduction

Membrane computing, also known as membrane systems or P systems, is a novel research of bioinspired computing initiated by Păun [1]. It seeks to discover novel biological computing models from the structure of biological cells as well as the cooperation of cells in tissues, organs, and populations of cells. Over the past years, there are three investigated P systems, cell-like P systems, tissue-like P systems, and neural-like P systems, including spiking neural P systems.

P systems have several characteristics: nondeterminism, programmability, extensibility, and readability [2]. Research shows that the some models of P systems present the same computing power as Turing machines and is more efficient to some extent [3]. Therefore, the analysis of computing power and computational efficiency of P systems is one of the important basic studies [4, 5]. Other studies are focused on the variation of P systems to solve optimization problems, including the variant of rules and structures [6, 7]. In addition, some intelligence techniques, such as evolutionary computation and fuzzy theory, is also introduced to the variant P systems in some specific optimization problems [8].

Because the parallel computation in membrane systems can avoid the increase in time consumption with the increase in the number of data points, the membrane systems are suitable for solving clustering problems [9]. There are a lot of interesting researches in variant P systems to solve clustering problems. Liu and Xue [10] proposed a new cluster splitting technique based on Hopfield networks and P systems. Liu et al. [11] presented an improved Apriori algorithm, named ECTPPT-Apriori, based on evolution-communication tissue-like P system with promoters and inhibitors. Peng et al. [12] developed an extended P system with active membranes, in which a modification differential evolution mechanism is used to find the optimal clustering centers in clustering problems. Peng et al. [13] introduced a multiobjective clustering framework using a tissue-like P system for solve fuzzy clustering problems. Wang et al. [14] proposed a new cell-like P system using a modified genetic algorithm to evolve the objects and using communication rules in the cell-like P system.

Image segmentation is an important part of image processing; it also has critical impact on the final quality of image analysis and subsequent tasks [15]. In the previous studies, the segmentation technique can be divided into region-based methods, edge-based methods, cluster-based methods, and threshold-based methods [16], in which the threshold methods can be classified into bilevel and multilevel threshold methods based on the number of clusters [17]. The region-based methods can obtain high segmentation quality but are sensitive to the parameters. The edge-based methods have high segmentation quality in different regions or targets and are also more sensitive to noise. The cluster-based methods are simple and easily implemented, but the clustering results rely on the number of clusters and feature selection in the colour space. The threshold-based methods are simple to computation, requiring no prior knowledge, but the continuity of the regions is not guaranteed due to the lack of space information [18]. So far, image segmentation has been used with wide recognized significance in machine vision, computer-aided diagnosis of the medical imaging, feature extraction, and analysis [19]. In this paper, the experiment on tested images is simple and easily implemented, and the application of image segmentation is not used in our works, so more details are not described in the following.

Because these segmentation methods mentioned above have their respective advantages and limitations, a lot of works have been done to find robust and optimum segmentation techniques [20]. The threshold technique is one of the most popular segmentation techniques which are based on the gray level of images. It is simplicity and easy implementation, which has lower computational complexity [21]. Li et al. [22] presented a novel thresholding extraction method based on variational mode decomposition (VMD). Zhao et al. [23] introduced a gradient-based adaptive particle swarm optimization (PSO) combined with improved external optimization (EO) to overcome being trapped into local optima in high-dimensional tasks. Wang et al. [24] designed a new P system with related interaction rules; the PSO mechanism is used to maximize entropy threshold. Tan et al. [25] proposed a hybrid clustering models using ensemble deep neural networks for skin lesion segmentation.

P systems are a class of distributed parallel computing models that can be used to improve the global search ability of PSO [26]. The commutation rules of chained P systems can be used to accelerate convergence of PSO. Compared with the crossover and mutation operation of DE, the partitioning information only can be used in the velocity updating of particles [27, 28]. Besides, although the neural networks have high quality for solving the optimization problems, the space and time consumption is too much, and the parallel computing model of P systems is also not executed on the neural networks. Therefore, a new variant of P systems based on PSO mechanism is proposed, which is named complex chained P (CCP) systems. And, the concepts of membrane, objects, and rules based on a special chained structure are introduced in the literature [29]. In this CCP system, two kinds of evolution rules in different chained membranes are introduced to enhance the global search ability. One of evolution rules is using the traditional and modified PSO mechanism to evolve the objects, and the partitioning information is introduced as the environmental factor to improve the clustering performance. Another is based on differential evolution (DE) to evolve the global chained objects in order to enhance the global search ability. Compared with genetic algorithm (GA) [30], the DE is simple and easily implemented, which has less predefined parameters and quick convergence speed. The communication rules for global objects between chained membranes are used to accelerate the convergence speed and avoid prematurity [31, 32]. At last, the CCP system is evaluated on eight benchmark clustering problems and eight tested images with the compared clustering and image segmentation techniques; the experimental results verify the validity and performance of proposed CCP.

The rest of this paper is organized as follows: the framework of the chained P systems is described in Section 2.  Section 3 gives more details about the complex chained P system for clustering problems, and the evolution rules and communication rules are described in this section. In order to verify the validity of CCP, some experiments which are conducted on benchmark clustering problems, are taken in Section 4.2. Furthermore, some tested images are used to evaluate the competitive performance of CCP in Section 4.3.  Section 5 provides some conclusions and outlines future research directions.

## 2. Chained P Systems

Some concepts can be briefly defined as follows. A special membrane is called *chained membrane*, which contains many objects based on a chained structure, and these objects are called *chained object.* The formal descriptions of the chained *P* system can be expressed as the following tuple [29].

(1) $$\begin{array}{c} \prod =\left(O,\mu ,w_{1},\cdots ,w_{m},R_{1},\cdots ,R_{m},\sigma_{\text{in}},\sigma_{\text{out}}\right), \end{array}$$

where

1. *O* is a finite nonempty alphabet, and the symbols are called chained objects.
2. *μ* is the membrane structure consisting of *m* membranes, which is composed of two parts: the structure of chained membranes and the structure of the whole chained P system.
3. *w*_1_, ⋯, *w*_*m*_ are the initial finite sets of chained objects; *w*_*i*_ represents the chained objects in membrane *i*, for 1 ≤ *i* ≤ *m*, *w*_*i*_ ∈ *O*.
4. *R*_1_, ⋯, *R*_*m*_ are the finite sets of chained rules, and *R*_*i*_ represents a finite set of chained rules in membrane *i*, for 1 ≤ *i* ≤ *m*, *R*_*i*_={*R*_*i*1_, *R*_*i*2_, ⋯*R*_*ir*_} which consists of *r* subrules that are executed on a special order. There are many chained rules on objects, for example, object addition rule, object subtraction rule, object crossover rule, and object variation rule.
5. *σ*_in_ is the input region or membrane in the chained P systems, which contains the initial objects in the systems.
6. *σ*_out_ is the output region or membrane in the chained P systems. If a certain chained rule cannot be executed in the chained P system, the computation process will be stopped and the computation results or objects will be transported in the output region or membrane.

## 3. Proposed Complex Chained P System for Clustering

The proposed chained P system based on evolutionary mechanism with a complex chained structure for clustering problems is presented in this section. First, the general framework of this complex chained P system (CCP) is described, and the basic membrane structure is given. Next, the evolution and communication mechanisms of chained P system are introduced in the CCP system. Finally, the computational process of the CCP system is described in the last parts.

### 3.1. General Framework of CCP System

The general framework of CCP is similar to that of the chained P systems, but the main differences are the membrane structure of whole P system and the evolution rules for chained objects. In complex chained P system, there are two kinds of chained membranes, chained membranes *σ*_1_ to *σ*_*m*_ and membranes *σ*_*m*+1_, which contains different evolution rules. These chained membranes are labelled from 1 to *m*+1. Respectively, the formal descriptions of the CCP system are as follows :

(2) $$\begin{array}{c} \prod =\left(O,\mu ,w_{1},\cdots ,w_{m},R_{1},\cdots ,R_{m},R_{1}^{\prime },\cdots ,R_{m+1}^{\prime },R_{m+1}^{*},\sigma_{\text{in}},\sigma_{\text{out}}\right), \end{array}$$

where

**Figure 1:**
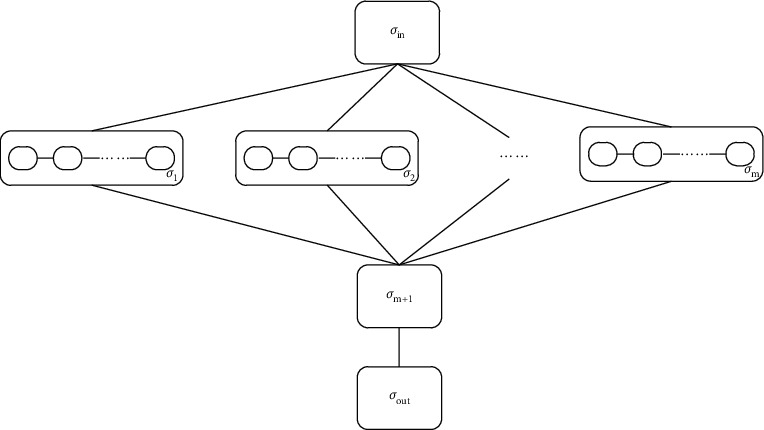
The membrane structure of CCP system.

1. *O* is a finite set of alphabets, which includes all chained objects or strings in the CCP systems.
2. *μ* is the membrane structure of CCP system consisting of *m*+3 membranes.
3. *w*_1_, ⋯, *w*_*m*_ are the multisets of the initial chained objects, with *w*_*i*_ ∈ *O*, for 1 ≤ *i* ≤ *m*, and *w*_*i*_ represents the chained objects.
4. *R*_1_, ⋯, *R*_*m*_ represent finite sets of evolution rules in chained membranes *σ*_1_ to *σ*_*m*_, *R*_*ir*_,  (1 ≤ *i* ≤ *m*) represent a set of evolution subrules in chained membrane *i*, and the number of the subrules are denoted by *r*. The subevolution rules are of the form: *R*_*ir*_={*u*⟶*v*}, with *u*, *v* ∈ *O*, which means that chained object *u* will be evolved to chained object *v*.
5. *R*_1_′, ⋯, *R*_*m*+1_′ represent the communication rules in chained membranes *σ*_1_ to *σ*_*m*+1_. The communication rules are of the form: *R*_*i*_′={*u*⟶(*v*, *in*_*j*_)}, with *u*, *v* ∈ *O*, for 1 ≤ *i* ≤ *m*+1, which means the chained object *u* in membrane *i* will be changed in object *v* and transported into membrane *j*.
6. *R*_*m*+1_^*∗*^ represents finite sets of evolution rules in chained membrane *σ*_*m*+1_. For two arbitrary chained objects *u* and *v*, *u*, *v* ∈ *O*, there are two kinds of evolution rules existing in the membranes can be executed: crossover rules and variation rules, *R*_*m*+1_^*∗*^={*R*_*m*+1,1_^*∗*^, *R*_*m*+1,2_^*∗*^}.
7. *σ*_in_ is the input region or membrane in the CCP system, which contains the initial objects of the whole system.
8. *σ*_out_ is the output region or membrane in the CCP systems. Once the computation is completed, the computation results or objects will be transported to the output region or membranes.  Figure 1 gives the membrane structure of the proposed CCP system.

In this proposed CCP, the chained objects are considered to be feasible solutions in the search space, and the partition obtained by the clustering technique is represented by *C*={*C*_*k*_}, for 1 ≤ *k* ≤ *K*. Thus, the *i*-th chained object *u*_*i*_ can be defined as *u*_*i*_=(**z**_*i*1_, **z**_*i*2_, ⋯, **z**_*iK*_), where *z*_*k*_,  (1 ≤ *k* ≤ *K*) represents the *k*-th cluster center, *K* is the number of the clusters, and the dimension of cluster center is denoted by *d*. At last, the best chained object that represents the best partitioning results in the system will be output to the *σ*_out_ when the computation is completed.

### 3.2. Evolution Rules on Chained Objects

There are two kinds of evolution rules on chained objects existing in the chained membranes, the evolution rules in chained membranes *σ*_1_ to *σ*_*m*_ and another evolution rules in chained membrane *σ*_*m*+1_.

#### 3.2.1. Evolution Rules in Chained Membranes *σ*_1_ to *σ*_*m*_

There are two kinds of evolution subrules in chained membranes *σ*_1_ to *σ*_*m*_, which are based on different velocity updating strategies for chained objects. Firstly, the particle swarm optimization (PSO) mechanism is used to evolve the chained objects, and the traditional velocity model of PSO is introduced to update the velocity of chained object. At *t*+1 time, the velocity formula of the *i*-th object *u*_*i*_ in *j*-th chained membrane *σ*_*j*_,  (1 ≤ *j* ≤ *m*) is determined by (3) in the following equation:

(3) $$\begin{array}{c} V_{i}\left(t+1\right)=wV_{i}\left(t\right)+c_{1}r_{1}\left(u_{i}^{\text{lbest}}\left(t\right)-u_{i}\left(t\right)\right)+c_{2}r_{2}\left(u_{j}^{\text{gbest}}\left(t\right)-u_{i}\left(t\right)\right), \end{array}$$

where the inertia weight is denoted by *w* and *c*_1_ and *c*_2_ represent the learning factor, which is usually restricted to 2. The iteration counter is denoted by *t*. *r*_1_ and *r*_2_ are two independent uniform random numbers. The local best of *u*_*i*_ in history is denoted by *u*_*i*_^lbest^, specially, the global best in *σ*_*j*_ is denoted by *u*_*j*_^gbest^.

Another velocity updating strategy for objects is based on environmental factors. At *t*+1 time, a modified velocity formula of EPSO is determined by (4) in the following equation:

(4) $$\begin{array}{c} V_{i}\left(t+1\right)=wV_{i}\left(t\right)+c_{1}r_{1}\left(u_{i}^{\text{lbest}}\left(t\right)-u_{i}\left(t\right)\right)+c_{2}r_{2}\left(u_{j}^{\text{gbest}}\left(t\right)-u_{i}\left(t\right)\right)+c_{3}r_{3}\left(E_{i}\left(t\right)-u_{i}\left(t\right)\right), \end{array}$$

where *c*_3_ represents a positive constant, *r*_3_ is a uniform random number, and *E*_*i*_(*t*) represents the environmental factor around to the object *u*_*i*_, which is based on the information of clusters. The partitioning information of cluster is changed dynamically through the evolution of objects, and the geometric center of the data point belonging to the corresponding cluster in the object *u*_*i*_ is used as the environment factor, where *E*_*i*_(*t*)={**e**_*i*1_(*t*), **e**_*i*2_(*t*), ⋯, **e**_*iK*_(*t*)}, for *k*=1,2, ⋯, *K*, which is given by (5) at *t* time in the following equation:

(5) $$\begin{array}{c} e_{ik}\left(t\right)=\frac{\sum_{x_{p}\in C_{k}}x_{p}}{N_{k}}, \end{array}$$

where *e*_*ik*_(*t*) represents the *k*-th cluster centroid of the environment factor *E*_*i*_, **x**_*p*_ represents the *p*-th data point in the dataset, for *p*=1,2, ⋯, *N*, *N* is the number of the data point in the datasets, and number of data points belonging to the corresponding cluster *C*_*k*_ is denoted by *N*_*k*_.

At *t*+1 time, the position formula of *u*_*i*_ is determined by (6) in the following equation:

(6) $$\begin{array}{c} u_{i}\left(t+1\right)=u_{i}\left(t\right)+V_{i}\left(t+1\right), \end{array}$$

and the local best of *u*_*i*_ at *t*+1 time is updated according to (7) in the following equation:

(7) $$\begin{array}{c} u_{i}^{\text{lbest}}\left(t+1\right)=\left\{\begin{array}{c} u_{i}\left(t+1\right),\;f\left(u_{i}\left(t+1\right)\right)<f\left(u_{i}^{\text{lbest}}\left(t\right)\right),\\ u_{i}^{\text{lbest}}\left(t\right),\;\text{otherwise,} \end{array}\right. \end{array}$$

where *f*(*u*) represents the fitness function of *u*_*i*_ for clustering problems, which can be defined by (8) in the following equation:

(8) $$\begin{array}{c} f\left(u_{i}\right)=\min\sum_{k=1}^{K}\sum_{j=1}^{N}\left\|x_{j}-z_{ik}\right\|. \end{array}$$

The purpose of clustering problems is to find a partitioning result C={*C*_1_, *C*_2_, ⋯, *C*_*K*_} obtained by techniques to minimize the values of fitness function according to equation (8). The global best *u*_*j*_^gbest^ at *t*+1 time is updated according to (7) in the following equation:

(9) $$\begin{array}{c} u_{j}^{\text{gbest}}\left(t+1\right)=\left\{\begin{array}{c} u_{i}^{\text{lbest}}\left(t+1\right),\;f\left(u_{i}^{\text{lbest}}\left(t+1\right)\right)<f\left(u_{j}^{\text{gbest}}\left(t\right)\right),\\ u_{j}^{\text{gbest}}\left(t\right),\;\;\text{otherwise.} \end{array}\right. \end{array}$$

At *t* time, a success rate of chained objects *u*_*i*_ is defined by the following equation:

(10) $$\begin{array}{c} S\left(u_{i},t\right)=\left\{\begin{array}{c} 1,\;if\;f\left(u_{i}^{\text{lbest}}\left(t\right)\right)<f\left(u_{i}^{\text{lbest}}\left(t-1\right)\right),\\ 0,\;if\;f\left(u_{i}^{\text{lbest}}\left(t\right)\right)=f\left(u_{i}^{\text{lbest}}\left(t-1\right)\right), \end{array}\right. \end{array}$$

where *S*(*u*_*i*_, *t*) represents the success rate of *u*_*i*_. The success rate in the chained membrane *σ*_*j*_, for 1 ≤ *j* ≤ *m*, is computed by (11) in the following equation:

(11) $$\begin{array}{c} P\left(\sigma_{j},t\right)=\frac{\sum_{i=1}^{n_{j}}s\left(u_{i},t\right)}{n_{j}}, \end{array}$$

where *n*_*j*_ represents the number of the chained object in chained membrane *σ*_*j*_ and *P*(*σ*_*j*_, *t*) is the success rate of *σ*_*j*_, *P*(*σ*_*j*_, *t*) ∈ [0,1], which means the improvement rate at last computation. In this study, a linearly increasing strategy is introduced to adjust the values of the inertia weight dynamically; the possible range is based on the mapping relationship of *P*(*σ*_*j*_, *t*), which is given by (12) in the following equation:

(12) $$\begin{array}{c} w\left(t\right)=\left(w_{\max}-w_{\min}\right)P\left(\sigma_{j},t\right)+w_{\min}, \end{array}$$

where *w*_min_and *w*_max_ represent the minimum and maximum of inertia weight and *w*(*t*) ∈ [*w*_min_, *w*_max_].

#### 3.2.2. Evolution Rules in Chained Membrane *σ*_*m*+1_

The evolution rules in chained membrane *σ*_*m*+1_ contain objects crossover rules and object variation rules, differential evolutionary (DE) approach is further used as the variant of evolution rules in *σ*_*m*+1_, and the mutation and crossover mechanism is also introduced to help the objects escape the local optima. At *t* time, the *i*-th chained object in *σ*_*m*+1_ is denoted by *u*_*i*_(*t*)=(*u*_*i*1_(*t*), *u*_*i*2_(*t*), ⋯, *u*_*i* *D*_(*t*)), where *D*=*k∗d* represents the dimension of the objects and *u*_*i*′_(*t*) and *u*_*i*^*∗*^_(*t*) are two randomly chained objects in *σ*_*m*+1_; the mutation operation is defined by (13) in the following equation:

(13) $$\begin{array}{c} v_{iy}\left(t\right)=u_{iy}\left(t\right)+F_{1y}*\left(u_{m+1y}^{\text{gbest}}\left(t\right)-u_{iy}\left(t\right)\right)+F_{2}*\left(u_{i\prime y}\left(t\right)-u_{i^{*}y}\left(t\right)\right),\;\text{for }y=1,2,\cdots ,D, \end{array}$$

where *v*_*i*_(*t*) represents the created donor, *u*_*m*+1_^gbest^(*t*) represents the best object in *σ*_*m*+1_, the scaling factor is denoted by *F*, and the value is given by *F*_1_=0.5 × (1+rand(0,1)), *F*_2_=0.5 × (1+rand(0,1)). The mutant *u*_*i*_′(*t*) comes from the created donor through crossover mechanism, which can be defined by

(14) $$\begin{array}{c} u_{iy}^{\prime }\left(t\right)=\left\{\begin{array}{c} v_{iy}\left(t\right),\;\text{if }{\text{rand}}_{i}\leq P_{c}\text{ or }y={\text{rand}}_{y},\\ u_{iy}\left(t\right),\;\text{otherwise}, \end{array}\right.\;\text{for }y=1,2,\cdots ,D, \end{array}$$

where the crossover rate is denoted by *P*_*c*_, it is a predefined constant within the range from 0 to 1, rand_*i*_ is an independent uniform random number, and rand_*y*_ is the random dimension from 1 to *D*. At *t*+1 time, the position formula of *u*_*i*_ is determined by

(15) $$\begin{array}{c} u_{i}\left(t+1\right)=\left\{\begin{array}{c} u_{i}^{\prime }\left(t\right),f\left(u_{i}^{\prime }\left(t\right)\right)<f\left(v_{i}\left(t\right)\right),\\ u_{i}\left(t\right),\;\text{otherwise.} \end{array}\right. \end{array}$$

### 3.3. Communication Rules in Chained Membranes

The communication rules of chained P system are used to enhance the cooperation between the chained membranes, which provide good foundation for exchange and sharing of the information. There are two kinds of communication rules in the CCP system:

1. *R*_*j*_′={*u*_*j*_^gbest^(*t*)⟶(*u*_*j*_(*t*), in_*m*+1_)}, for *j*=1,2, ⋯, *m*. At *t* time, the global best *u*_*j*_^gbest^ in *σ*_*j*_ is sent to the chained membrane *σ*_*m*+1_ and transformed into the *j*-th object *u*_*j*_. At each iterative computation, the chained membrane *σ*_*m*+1_ only contains *m* objects that come from the chained membranes *σ*_1_ to *σ*_*m*_.
2. *R*_*m*+1_′={*u*_*m*+1_^gbest^(*t*)⟶(*u*_*j*_^gbest^(*t*), in_*j*_)}, for *j*=1,2, ⋯, *m*. At *t* time, the global best *u*_*m*+1_^gbest^ in *σ*_*m*+1_ is transported to the chained membranes *σ*_1_ to *σ*_*m*_, which is considered to be the global best *u*_*j*_^gbest^ in chained membrane *σ*_*j*_. Meanwhile, the global best *u*_*m*+1_^gbest^ in membrane *σ*_*m*+1_ is sent to the output membrane *σ*_out_, which is viewed as the best object or the final computation results of the CCP at *t* time.

### 3.4. Computation Process of CCP

#### 3.4.1. Initialization

The input membrane *σ*_in_contains all initial objects in the P system, denoted by *Q*=*m∗n*. The position of chained object is randomly initialized in the search space. After initialization, the chained objects in the membrane *σ*_in_ will be transported to the chained membrane *σ*_1_ to *σ*_*m*_, and each chained membrane contains *n* objects.

#### 3.4.2. Evolution in Chained Membranes *σ*_1_ to *σ*_*m*_

The evolution rules on chained objects are used to completed the evolution process according to the equations (3), (4),and (6) in chained membrane *σ*_*j*_,  (1 ≤ *j* ≤ *m*). And, the selection for velocity formula is based on a random strategy. The local best and global best in *σ*_*j*_ are updated through the equations (7) and (9).

#### 3.4.3. Communication between Chained Membranes

The first kinds of communication rules are used to transport the global best in the *σ*_*j*_,  (1 ≤ *j* ≤ *m*) to the chained membrane *σ*_*m*_.

#### 3.4.4. Evolution Rules in Chained Membranes *σ*_*m*+1_

The evolution rules on chained objects are used to completed the evolution process according to the equations (13) and (14) in chained membrane *σ*_*m*+1_, and global best in *σ*_*m*+1_ is updated by the equation (15).

#### 3.4.5. Communication between Chained Membranes

The second kinds of communication rules are used to transport the global best in *σ*_*m*_ to each chained membrane *σ*_*j*_,  (1 ≤ *j* ≤ *m*).

#### 3.4.6. Halting and Output

The evolution and communication will be implemented repeatedly with an iterative form during the computation process. The termination criterion of the system is stopped to whether the maximum number of iterations or computation is reached. When the system halts, the output membrane *σ*_out_ will send the global best to the environment, and this object is regarded as the final computing results of CCP system.

## 4. Experimental Analysis of Clustering Problems

In this section, the feasibility and effectiveness of the proposed CCP will be demonstrated through the experimental analysis. The datasets of the experiment are introduced first, and the artificial dataset from the previous studies [33] is used to tune the parameters in CCP. Eight real-life datasets from UCI machine leaning repository [34] is used to compare the performance with currently existing clustering approaches. All clustering approaches, including CCP, are implemented on MATLAB 2016b, and all the experiments are conducted on a DELL desktop computer with an Intel 4.00 GHz i7-8550U processor and 8 GB of RAM in a Windows 10 environment.

### 4.1. Parameter Setting

The numbers of chained membranes have important influences on the performance of CCP. Therefore, four artificial datasets [33], *Data_5_2*, *Data_9_2*, *Size5*, and *Square4*, are used to tune this parameter in order to ensure the equity of the experiment. The details of the four artificial datasets are given in Table 1.

Different CCP systems with different degrees of *m*=4, *m*=5, *m*=6 [12] are used to evaluate the effects of the number of the chained membranes. The maximum of iterations is set to *t*_max_=100, and the positive constant *c*_3_ is a random number which distributed to 0.6 and 3. The lower and upper limits of the inertia weight are set to *w*_min_=0.4 and *w*_max_=1.2. The mutation probability *P*_*c*_ is randomly generated from 0.2 to 0.8. Other parameters which are not tested in this experiment will be maintained the same values for the fairness of the comparison experiments. And, the number of independent running is set to 30 to eliminate the effect of the random factors. The values of mean and SD of the fitness function obtained by CCP system with different degrees are reported in Table 2.

Table 2 reports the clustering results of the CCP system with different degrees on four artificial datasets. The best values of mean and SD for each dataset are given in bold. These results show that the mean and the SD values obtained by the CCP system when *m*=10 are the best among these different systems.

### 4.2. Clustering Problems

The performance of the CCP is compared with four clustering approaches that have been reported in the literature to further evaluate the effectiveness, such as standard particle swarm optimization (PSO) [35], differential evolution (DE) [36], environment particle swarm optimization (EPSO) [37], and adaptive particle swarm optimization (APSO) [38]. The comparison experiments are conducted on eight real-life datasets from the UCI machine learning repository, *Iris*, *Newthyroid*, *Seeds*, *Diabetes*, *Yeast*, *Glass*, *CMC*, and *Lung Cancer*. More details about these datasets are presented in Table 3.

In these compared clustering approaches, the crossover probability *P*_*c*_ of the DE approach is randomly generated from 0.2 to 0.8. An environment factor based on the clustering information is embedded in the velocity updating model of EPSO approach, and the learning factor *c*_3_ is randomly generated from 0.6 to 3. A nonlinear regressive function of APSO based on the population diversity is used to the adjustment formula of the inertia weight, *L* represents a predefined constant, and *c* is a predefined constant. All adjustable parameters in these clustering approaches are set to the appropriate values which are reported in the respective publications from Table 4.

Each clustering approach, including CCP, ran for 50 times for each dataset to eliminate the effects of random factors. Simple statistics including worst values (Worst), best value (Best), Mean, and SD of fitness function according to (8) are used in the experiments as the evaluation criteria of clustering results. The experimental environment is the same for all comparative clustering approaches.

**Figure 2:**
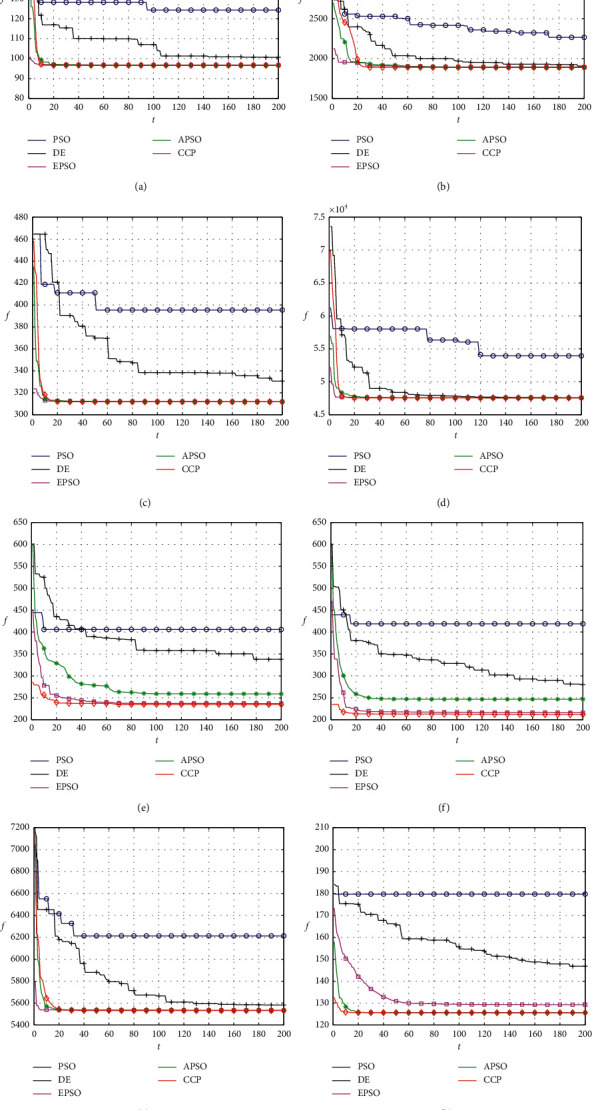
Convergence of the approaches on each of the eight real-life datasets. (a) *Iris*, (b) *Newthyroid*, (c) *Seeds*, (d) *Diabetes*, (e) *Yeast*, (f) *Glass*, (g) *CMC*, and (h) *Lung Cancer*.

Figure 2 shows the convergence of these clustering approaches on the eight test datasets for typical runs of these approaches. The fitness value obtained by CCP declines faster at the beginning of the evolution process and then obtains fine convergence for each dataset. The values of the fitness function of PSO and DE decrease slowly at the beginning of the evolution process and do not apparently have better convergence performance than other approaches. Although EPSO and APSO show better performance than the above clustering approaches, they are also easily trapped into local optima, as shown in Figures 2(e), 2(f), and 2(h). Therefore, CCP has better convergence speed and higher clustering quality than the comparative approaches for all these datasets, as shown in Figure 2.

Simple statistics of the fitness function values of these clustering approaches on these datasets are reported in Table 5. Results in Table 5 show that CCP has the overall best performance on these eight test datasets. Due to the characteristics of the test datasets, some clustering approaches performed better on some specific datasets with smaller SD, but the performance of CCP on these ten datasets is all considered comparable.  Table 6 provides the average computation time in seconds taken by each of the five clustering techniques when running 50 times on each of the datasets. It can be seen from Table 6 that the proposed CCP has a larger average computation time as compared with PSO, DE, EPSO, and APSO. The evolution process in the chained membranes is time-consuming so that CCP takes more time than other techniques does.

In order to evaluate the clustering performance of these compared clustering approaches, clustering accuracy (CA) is used to evaluate the quality of clustering results obtained by the clustering techniques; the overall accuracy of partitioning results is defined by

(16) $$\begin{array}{c} \text{CA}=\frac{\sum_{k=1}^{K}\max_{l=1}^{K}\left|C_{k}\cap C_{l}\right|}{N}, \end{array}$$

where |*C*_*k*_∩*C*_*l*_| represents the number of the data points in both belonging to actual cluster *k* and partitioning cluster *l*, for *k*,  *l*=1,2, ⋯, *K*. The simple statistics of clustering approaches on eight datasets are reported in Table 7. It can be seen that CCP has overall better performance on these datasets. Although some approaches show a better performance on some specific datasets, the performance of CCP on these specific datasets is also considered compared from Table 7.

### 4.3. Proposed CCP for Image Segmentation

**Figure 3:**
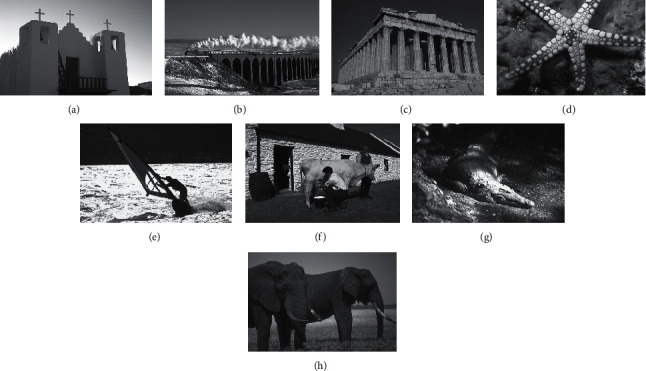
Benchmark tested images. (a) Church. (b) Train. (c) Roman. (d) Starfish. (e) Surfer II. (f) Cow. (g) Crocodile. (h) Elephant.

In this section, some typical experiment and analysis for tested image are proposed to evaluate the segmentation performance of this proposed CCP. These tested images are used in the previous studies and researches, which are provided from the Berkeley segmentation dataset and benchmark [39]. The size of the tested image is 481 × 321; Figure 3 gives the original image of these tested images.

OSTU proposed by Qtsu [40] is one of most popular segmentation methods, and it has been used to determine whether the optimal threshold method can give a satisfactory segmentation results. The following discriminant criterion measure of OSTU can be described as follows: An image contains *N* pixels from 0 to *L*, usually *L* is set to 255, need to segmented in *M*+1 clusters. Thus, *M* thresholds {*t*_1_, *t*_2_, ⋯, *t*_*M*_} are needed that divided the original image. The number of *i*-th gray level pixels or frequencies is denoted by *h*(*i*), PR_*i*_ represents the probability of *i*-th gray level pixel in the image,PR_*i*_=*h*(*i*)/*N*, where *N*=∑_*i*=0_^*L*^*h*(*i*). The optimal thresholds {*t*_1_, *t*_2_, ⋯, *t*_*M*_} are determined by the following equations:

(17) $$\begin{array}{c} \left\{t_{1}^{*},t_{2}^{*},\cdots ,t_{M}^{*}\right\}=\arg\max\left\{\sigma_{B}^{2}\left(t_{1},t_{2},\cdots ,t_{M}\right)\right\}, \end{array}$$

(18) $$\begin{array}{c} \sigma_{B}^{2}={\sum_{k=1}^{M}w_{k}*\left(u_{k}-u_{t_{k}^{*}}\right)}^{2}, \end{array}$$

where *w*_*k*_^2^=∑_*i*∈*C*_*k*__PR_*i*_, *u*_*k*_^2^=∑_*i*∈*C*_*k*__*iPR*_*i*_/*w*_*k*_, for *k*=1,2, ⋯, *M*.

**Figure 4:**
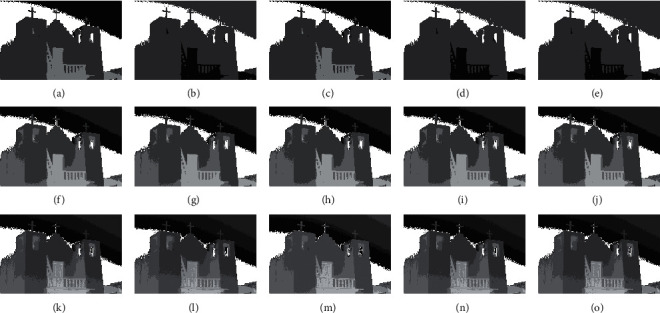
Segmented images of church at different thresholds calculated by all approaches: (a) PSO (Th = 3), (b) DE (Th = 3), (c) EPSO (Th = 3), (d) APSO (Th = 3), (e) CCP (Th = 3), (f) PSO (Th = 5), (g) DE (Th = 5), (h) EPSO (Th = 5), (i) APSO (Th = 5), (j) CCP (Th = 5), (k) PSO (Th = 8), (l) DE (Th = 8), (m) EPSO (Th = 8), (n) APSO (Th = 8), (o) CCP (Th = 8).

**Figure 5:**
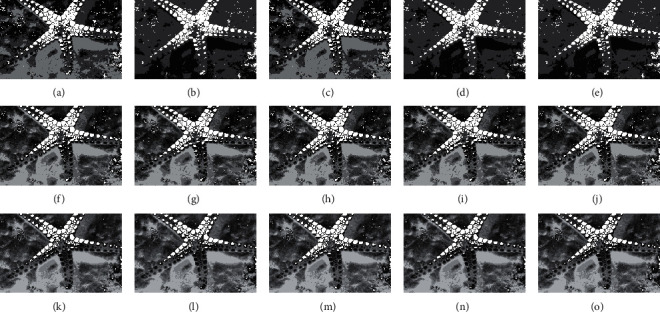
Segmented images of starfish at different thresholds calculated by all approaches: (a) PSO (Th = 3), (b) DE (Th = 3), (c) EPSO (Th = 3), (d) APSO (Th = 3), (e) CCP (Th = 3), (f) PSO (Th = 5), (g) DE (Th = 5), (h) EPSO (Th = 5), (i) APSO (Th = 5), (j) CCP (Th = 5), (k) PSO (Th = 8), (l) DE (Th = 8), (m) EPSO (Th = 8), (n) APSO (Th = 8), (o) CCP (Th = 8).

**Figure 6:**
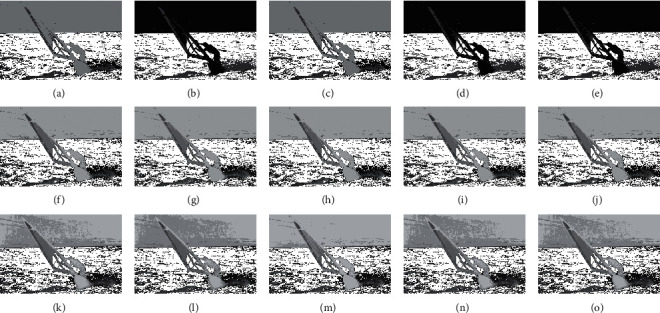
Segmented images of Surfer II at different thresholds calculated by all approaches: (a) PSO (Th = 3), (b) DE (Th = 3), (c) EPSO (Th = 3), (d) APSO (Th = 3), (e) CCP (Th = 3), (f) PSO (Th = 5), (g) DE (Th = 5), (h) EPSO (Th = 5), (i) APSO (Th = 5), (j) CCP (Th = 5), (k) PSO (Th = 8), (l) DE (Th = 8), (m) EPSO (Th = 8), (n) APSO (Th = 8), (o) CCP (Th = 8).

**Figure 7:**
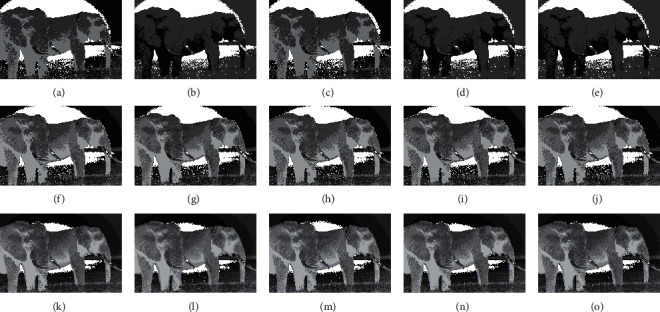
Segmented images of elephant at different thresholds calculated by all approaches: (a) PSO (Th = 3), (b) DE (Th = 3), (c) EPSO (Th = 3), (d) APSO (Th = 3), (e) CCP (Th = 3), (f) PSO (Th = 5), (g) DE (Th = 5), (h) EPSO (Th = 5), (i) APSO (Th = 5), (j) CCP (Th = 5), (k) PSO (Th = 8), (l) DE (Th = 8), (m) EPSO (Th = 8), (n) APSO (Th = 8), (o) CCP (Th = 8).

The compared experiments are performed on the test images with a different number of thresholds, *M*=3, *M*=5, and *M*=8 [41], to evaluate the performance of the CCP in both low- and high-dimensional multilevel thresholding problems. And, the proposed CCP is compared with PSO, DE, EPSO, and APSO approaches as mentation above. The purpose of image segmentation is to find a set of thresholds {*t*_1_, *t*_2_, ⋯, *t*_*M*_} to maximize the values of Ostu's function according to equation (17). Figures 4–7 give the segmented results on tested images with a different number of thresholds.

Figures 4–7 provide the segmented images on church, starfish, surfer II, and elephants obtained by the compared techniques. It can be observed that the segmented quality has been improved with the increasing number of thresholds. And, CCP has a better performance than others on these tested images. Respectively, the segmented images obtained by APSO and CCP achieve better consistency than those by PSO, DE, and EPSO when Th=8. The gray-level histogram is often regarded as a kind of distributions to determine the thresholds for the image segmentation [42]. And, the peak value of the histogram is one of the important factors that affect the segmentation accuracy. Therefore, the thresholds of the compared approaches on church and elephant images are shown in gray-level histogram as follows.

**Figure 8:**
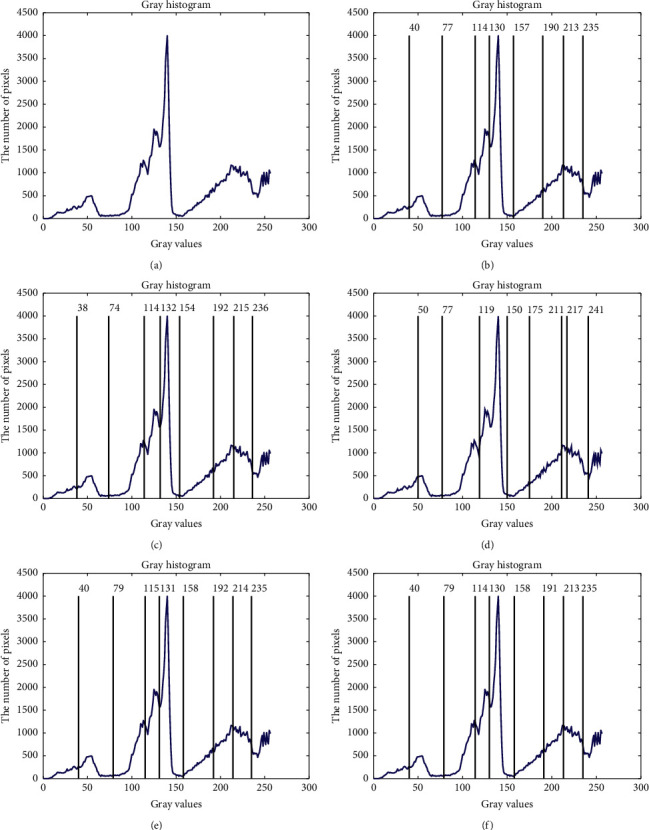
Segmented images of church at 8 thresholds level calculated by all approaches and their respective gray histogram. (a) Church, (b) PSO, (c) DE, (d) EPSO, (e) APSO, and (f) CCP.

**Figure 9:**
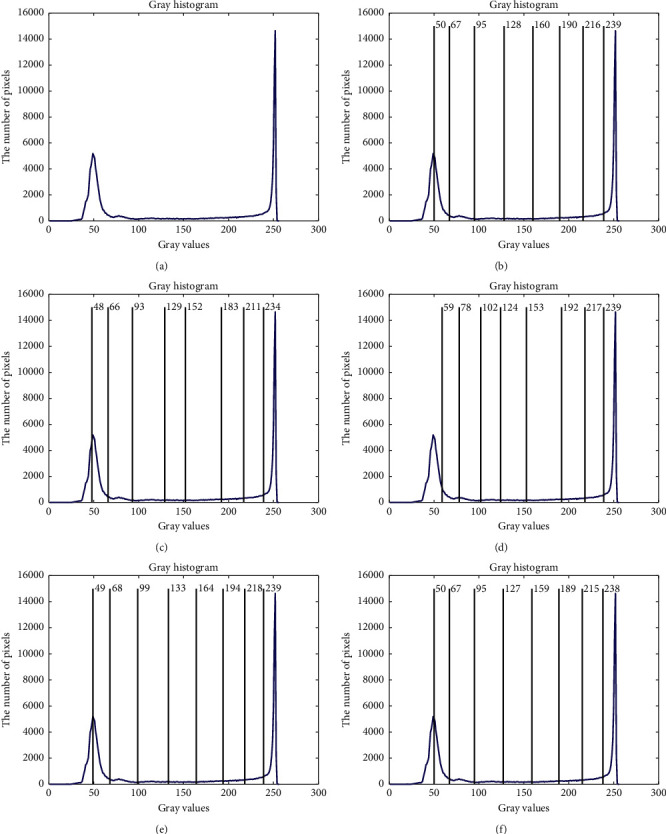
Segmented images of elephant at 8 thresholds level calculated by all approaches and their respective gray histogram. (a) Elephant, (b) PSO, (c) DE, (d) EPSO, (e) APSO, and (f) CCP.

Figures 8 and 9 show the segmented results of church and elephant at 8 thresholds level obtained by CCP and compared approaches. The optimal thresholds values of CCP with {40, 79, 114, 130, 158, 191, 213, 235} is similar to those of the PSO with {40, 77, 114, 130, 157, 190, 213, 235} on church images and also similar to those of the elephant image. From these figures, it can be seen that the optimal thresholds values by DE and EPSO are very different from those by PSO, APSO, and CCP in most cases. Because the segmentation results depend on the information of classes according to the thresholds level, the optimal thresholds of EPSO have statistical difference with others. Furthermore, it is not difficult to find that the selected optimal thresholds heavily depend on the objective function that is chosen. Besides EPSO, other threshold selection approaches, including the CCP approach, can segment the test images more reasonably, as shown in Figures 4–7.  Table 8 provides the mean and S of Ostu's function with 3, 5, and 8 thresholds achieved by all compared approaches in 50 run times.

The simple statistics of compared approaches on tested images are reported in Table 8. The best values of mean and SD for each image are highlighted, and it is not hard to see that the CCP system is able to find the best values. Because the PSO, DE, EPSO, and APSO are not specially techniques for image segmentation, some segmented approaches based on multilevel threshold, whale optimation algorithm (WOA) [43], gray wolf optimizer (GWO) [44], whale optimization algorithm based on thresholding heuristic (WOA-TH) [41], and gray wolf optimizer based on thresholding heuristic (GWO-TH) [41], are used to evaluate the clustering effectiveness of CCP. The maximum number of iterations is set to 2200, 3000, and 3600 with the number of thresholds being 3, 5, and 8. The mean and SD of Ostu's function are obtained by the 100 run times to avoid the effects of random factors.

Table 9 provides the values of mean and SD obtained by different segmented approaches on tested images. Obviously, traditional approaches are easily trapped into local optima, and WOA and GWO have worse segmented performance than that of the others. And, the thresholding heuristic has finetuned the best thresholds to enhance the global search ability of WOA and GWO. It also can be observed that the CCP has better and stable performance than compared segmented approaches on the tested images.

## 5. Conclusions

A complex chained P system (CCP) is proposed for solving clustering problems, which combines complex chained P systems and evolution mechanisms, including PSO and DE mechanisms. Two kinds of evolution rules for objects in different chained membranes are introduced to enhance the global search ability of PSO. One of evolution rules contain two subevolution rules, which are based on traditional and modified PSO techniques. The partitioning information as environmental factor is introduced to improve the clustering performance of PSO. Another is based on the DE mechanism to evolve the global chained objects in the chained membrane *m*+1 to enhance the global search ability. In addition, two kinds of communication rules in the chained P systems are defined to enhance the cooperation between chained membranes and avoid prematurity. In order to verify the validity and the performance of CCP, this proposed system is evaluated on eight benchmark clustering problems from the UCI machine learning repository as compared with four developed clustering approaches. Furthermore, eight tested images which from the Berkeley segmentation image databases BSDS300 are used to further evaluate the performance of CCP compared to four existing segmentation techniques. These experimental results verify the validity and performance of this proposed CCP.

P systems, as parallel computing models, are highly effective and efficient in solving optimization problems with linear or polynomial complexity. These parallel computing models based on evolution mechanisms provide new ways for solving clustering problems. The extended clustering chained P system uses the chained P system as the computation structure, and the communication rules between chained membranes are single directional. Although these single directional communication rules are simple and easy to implement, bidirectional communication rules may be introduced in future studies to further accelerate the convergence and improve the diversity of populations. Some more complicated communication structures between different membranes may be used in future studies to improve the performance of the approach. Furthermore, the experiments only used small datasets from the artificial datasets and the UCI Machine Learning Repository, and the proposed approach may have some limitations on high dimensional and large datasets. Future studies may test the effectiveness of CCP using large datasets. Balancing the local and global search abilities is also a hard problem to resolve in the future studies. Future studies may also focus on extended P systems based on tissue-like P systems and other bioinspired computing models. More works are needed to apply these extended membrane systems to solve automatic and multiobjective clustering problems.

## Figures and Tables

**Table 1:** Properties of the artificial datasets.

Datasets	Data	Feature	Class
*Data_5_2*	250	2	5
*Data_9_2*	900	2	9
*Size_5*	1000	2	4
*Square4*	1000	2	4

**Table 2:** Performance of CCP with different degrees.

Datasets	*m*=4		*m*=5		*m*=10	
	Mean	SD	Mean	SD	Mean	SD
*Data_5_2*	326.5071	0.0301	326.4696	0.0123	**326.4641**	**0.0110**
*Data_9_2*	590.8587	0.0624	590.7589	0.0463	**590.7413**	**0.0287**
*Size_5*	2493.1450	4.8048	2491.9680	0.0789	**2491.9020**	**0.0565**
*Square4*	2367.6443	0.0317	2367.6052	0.0260	**2367.5917**	**0.0141**

**Table 3:** Properties of the UCI datasets.

Datasets	Data	Feature	Class
*Iris*	150	4	3
*Newthyroid*	215	5	3
*Seeds*	210	7	3
*Diabetes*	768	8	2
*Yeast*	1484	8	10
*Glass*	214	9	6
*CMC*	1473	9	3
*Lung Cancer*	32	26	3

**Table 4:** Parameter setting of the comparative clustering approaches used in the experiments.

Parameters	PSO	DE	EPSO	APSO	CCP
Population (*Q*)	100	100	100	100	100
*t*_max_	200	200	200	200	200
*c*_1_,*c*_2_	2,2	N	2,2	N	2,2
*c*_3_	N	N	(0.6, 3)	N	(0.6, 3)
*r*_1_,*r*_2_	(0,1)	N	(0, 1)	N	(0, 1)
*r*_3_	N	N	(0, 1)	N	N
(*w*_min_,*w*_max_)	1	N	(0.4, 0.6)	N	(0.4, 1.2)
*P*_*c*_	N	(0.2, 0.8)	N	N	(0.2, 0.8)
*L*	N	N	N	2.1	N
*c*	N	N	N	2	N
*m*	N	N	N	N	10

**Table 5:** Performance of the comparative clustering approaches on the eight real-life datasets.

Datasets	Statistics	Clustering approaches				
		PSO	DE	EPSO	APSO	CCP
*Iris*	Worst	153.8256	109.9969	96.8183	96.7113	**96.6555**
	Best	114.6361	101.9888	96.7063	96.6672	**96.6555**
	Mean	133.4267	105.2555	96.7431	96.6863	**96.6555**
	S.D.	8.7150	2.3362	0.0374	0.0093	**1.23*E*** **−** **13**

*Newthyroid*	Worst	2400.0152	1977.3729	1943.6091	1908.2618	**1895.9965**
	Best	2040.0058	1924.2365	1887.7578	1885.4950	**1866.5183**
	Mean	2271.0529	1950.8237	1903.7699	1897.2114	**1888.1277**
	S.D.	91.1317	14.0217	11.1225	**5.0863**	7.5021

*Seeds*	Worst	452.8039	353.1875	312.1602	311.9663	**311.7978**
	Best	384.2174	333.9361	311.8824	311.8493	**311.7978**
	Mean	422.1335	342.6432	312.0224	311.9148	**311.7978**
	S.D.	23.0106	5.4078	0.0715	0.0257	**4.27*E*** **−** **07**

*Diabetes*	Worst	59885.0835	47911.2714	47629.8621	47590.4655	**47561.1362**
	Best	51628.1083	47693.6403	47585.4012	47561.6124	**47561.1262**
	Mean	54535.4335	47779.1879	47607.6176	47573.1668	**47561.1262**
	S.D.	2147.4319	73.2248	11.8570	6.9363	**3.79*E*** **−** **08**

*Yeast*	Worst	430.0923	378.2874	248.9934	287.4479	**245.0999**
	Best	391.0852	348.2327	236.9439	256.7699	**235.3785**
	Mean	407.6461	367.3743	242.7818	271.6039	**239.6706**
	S.D.	10.9776	7.2050	3.3082	8.0848	**2.4396**

*Glass*	Worst	451.0029	334.2308	226.0186	253.7243	**213.4793**
	Best	328.2842	292.1006	212.6117	210.8631	**212.1629**
	Mean	418.7840	314.2182	216.2731	230.8009	**212.9308**
	S.D.	31.2743	8.9158	3.5290	14.8939	**0.3110**

*CMC*	Worst	6852.7697	5720.0002	5537.8142	5535.5675	**5532.3096**
	Best	6335.6713	5628.6990	5535.5853	5533.1403	**5532.1857**
	Mean	6551.6558	5683.0978	5536.8242	5534.1784	**5532.1913**
	S.D.	174.0443	28.1480	0.5543	0.5962	**0.0278**

*Lung Cancer*	Worst	170.2152	153.9227	127.4346	125.6464	**125.6485**
	Best	184.9632	159.9095	138.4711	126.3331	**125.6685**
	Mean	178.8476	157.0802	133.1676	125.7308	**125.6609**
	S.D.	4.1542	1.6051	3.3466	0.1595	**0.0052**

**Table 6:** Average computation time in seconds over 50 runs.

Datasets	Clustering approaches				
	PSO	DE	EPSO	APSO	KEPSO-CCP
*Iris*	2.4630	2.7315	3.0608	2.6708	3.3833
*Newthyroid*	2.4785	2.8251	3.3350	2.8360	3.6576
*Seeds*	2.4263	2.7072	3.2817	2.8446	3.5799
*Diabetes*	2.7341	3.1116	4.9156	3.2315	5.2833
*Yeast*	5.1674	5.2355	9.8255	5.6831	10.3316
*Glass*	2.6526	3.0069	3.6378	2.9946	4.0859
*CMC*	3.7550	4.0391	8.0583	3.9940	8.1726
*Lung Cancer*	2.3451	2.7382	2.8267	2.7655	3.2006

**Table 7:** Performance of the comparative clustering approaches on the eight real-life datasets.

Datasets	Statistics	Clustering approaches				
		PSO	DE	EPSO	APSO	CCP
*Iris*	Worst	0.6667	0.8933	0.8933	**0.9000**	0.8667
	Best	0.9267	0.9000	0.9000	0.9000	**0.9600**
	Mean	0.8077	0.8983	0.8997	0.9000	**0.9013**
	SD	0.0904	0.0027	0.0015	**1.03*E*** **−** **17**	0.0248

*Newthyroid*	Worst	0.6977	0.7488	0.7721	0.7721	**0.7767**
	Best	**0.8372**	0.8093	0.8047	0.8047	0.8047
	Mean	0.7391	0.7777	0.8000	0.8000	**0.8014**
	SD	0.0337	0.0187	0.0114	0.0114	**0.0080**

*Seeds*	Worst	0.6476	0.8477	0.8952	0.8952	**0.8952**
	Best	0.8953	**0.9143**	0.8952	0.8952	0.8953
	Mean	0.8367	0.8814	0.8952	0.8952	**0.8952**
	SD	0.0850	0.0207	1.14*E* − 16	1.14*E* − 16	**1.14*E*** **−** **16**

*Diabetes*	Worst	0.6510	0.6510	0.6510	0.6510	**0.6510**
	Best	0.6510	0.6510	0.6510	0.6510	**0.6654**
	Mean	0.6510	0.6510	0.6510	0.6510	**0.6518**
	SD	1.14*E* − 16	1.14*E* – 16	1.14*E* − 16	**1.14*E*** **−** **16**	0.0032

*Yeast*	Worst	0.3120	0.3309	0.4508	0.3942	**0.4683**
	Best	0.3376	0.4016	0.5370	0.4569	**0.5418**
	Mean	0.3171	0.3640	0.4965	0.4377	**0.5200**
	SD	**0.0057**	0.0248	0.0275	0.0134	0.0152

*Glass*	Worst	0.3551	0.4720	0.5607	0.5093	**0.5841**
	Best	0.4813	0.5234	**0.5981**	0.5841	0.5841
	Mean	0.4140	0.5021	0.5834	0.5486	**0.5841**
	SD	0.0444	0.0144	0.0122	0.0267	**1.14*E*** **−** **16**

*CMC*	Worst	0.4332	0.4358	0.4515	**0.4562**	0.4515
	Best	0.4630	**0.4582**	0.4569	0.4562	0.4569
	Mean	0.4510	0.4492	0.4556	0.4562	**0.4562**
	SD	0.0087	0.0074	0.0016	**5.66*E*** **−** **17**	0.0012

*Lung Cancer*	Worst	0.4062	0.4063	0.5313	0.4063	**0.5625**
	Best	0.5625	0.5000	0.5625	**0.6250**	0.5625
	Mean	0.4641	0.4281	0.5578	0.5188	**0.5625**
	SD	0.0433	0.0250	0.0114	0.0644	**1.28*E*** **−** **16**

**Table 8:** The values of Ostu's function with a different number of thresholds calculated by all approaches.

Images	Th	P	Segmentation approaches				
			PSO	DE	EPSO	APSO	CCP
Church	3	Mean	3276.8367	3276.837	3276.7685	3276.8367	**3276.8367**
		SD	1.87*E* − 12	1.87*E* − 12	0.155831	1.87*E* − 12	**0.00*E*** **+** **00**
	5	Mean	3381.9107	3381.4923	3380.4929	3381.9236	**3381.9246**
		SD	0.0171	0.2245	1.1240	0.0046	**9.25*E*** **−** **13**
	8	Mean	3422.5621	3420.9578	3416.2082	3420.7834	**3423.2006**
		SD	2.6579	**0.9181**	3.5574	2.86725	1.2323

Train	3	Mean	2606.5951	2606.5951	2606.5623	2606.5951	**2606.5951**
		SD	**4.67*E*** **−** **13**	4.67*E* − 13	0.0500	4.67*E* − 13	**4.67*E*** **−** **13**
	5	Mean	2736.2257	2735.3181	2735.2676	2735.6328	**2736.2331**
		SD	0.0135	0.5241	0.6743	2.6787	**9.33*E*** **−** **13**
	8	Mean	2790.3435	2788.0338	2786.9829	2790.4068	**2790.4461**
		SD	0.0913	0.5724	2.5511	0.0897	**0.0389**

Roman	3	Mean	2142.3440	2142.3440	2142.2752	2142.3440	**2142.3440**
		SD	9.33*E* − 13	9.33*E* − 13	0.1073	9.33*E* − 13	**1.39*E*** **−** **13**
	5	Mean	2221.3026	2220.8465	2220.5998	2221.3051	**2221.3056**
		SD	0.0047	0.2476	0.4636	0.0012	**1.67*E*** **−** **13**
	8	Mean	2251.7218	2250.5766	2249.7117	2251.7258	**2251.7325**
		SD	0.0592	0.4211	1.2147	0.0673	**0.0573**

Starfish	3	Mean	2784.2272	2784.2272	2784.0795	2784.2272	**2784.2272**
		SD	1.4*E* − 12	1.4*E* − 12	0.1267	1.4*E* − 12	**9.25*E*** **−** **13**
	5	Mean	2916.2557	2915.6466	2915.5862	2916.2726	**2916.2730**
		SD	0.0240	0.3693	0.6716	0.0010	**0.0009**
	8	Mean	2973.9976	2971.8186	2971.2207	2974.0391	**2974.0829**
		SD	0.0404	0.7603	1.8786	0.0942	**0.0151**

Surfer II	3	Mean	7953.4181	7953.4181	7953.3489	7953.4181	**7953.4181**
		SD	2.80*E* − 12	2.80*E* **−** 12	0.0718	**2.78*E*** **−** **12**	2.80*E* − 12
	5	Mean	8025.5880	8025.1056	8024.6160	8025.6045	**8025.6055**
		SD	0.0147	0.2639	1.2232	0.0031	**0.0019**
	8	Mean	8055.9754	8054.3387	8052.1159	8056.0443	**8056.0530**
		SD	0.0505	0.5497	2.2741	0.0361	**0.0189**

Cow	3	Mean	3858.2202	3858.2202	3858.1872	3858.2200	**3858.2202**
		SD	0.00*E* + 00	0.00*E* + 00	0.0886	0.0009	**0.00*E*** **+** **00**
	5	Mean	3965.2806	3964.3722	3964.0770	3965.2925	**3965.2932**
		SD	0.0196	0.4531	1.2480	0.0032	**4.67*E*** **−** **13**
	8	Mean	4028.8447	4026.0490	4024.4213	4028.9299	**4028.9450**
		SD	0.0758	0.8635	2.6447	0.0548	**0.0107**

Crocodile	3	Mean	3155.4359	3155.4343	3155.3468	3155.4359	**3155.4359**
		SD	9.33*E* − 13	0.0069	0.0969	9.33*E* **−** 13	**9.25*E*** **−** **13**
	5	Mean	3291.4296	3290.6065	3290.4743	3291.4263	**3291.4407**
		SD	0.0163	0.4943	1.0594	0.0323	**0.00*E*** **+** **00**
	8	Mean	3348.6314	3346.4809	3345.8010	3348.6425	**3348.7305**
		SD	0.0719	0.6380	2.0889	0.1033	**0.0168**

Elephants	3	Mean	1626.7205	1626.7205	1626.6710	1626.7198	**1626.7205**
		SD	**0.00*E*** **+** **00**	0.00*E* + 00	0.1220	0.0035	0.00*E* + 00**0**
	5	Mean	1695.8663	1695.5298	1695.2335	1695.8663	**1695.8676**
		SD	0.0039	0.1415	0.8755	0.0023	**0.0005**
	8	Mean	1728.1706	1726.9276	1726.1534	1728.0408	**1728.2503**
		SD	0.0592	0.3931	1.5228	0.6764	**0.0232**

**Table 9:** The values of Ostu's function with a different number of thresholds calculated by segmentation approaches.

Images	Th	P	Segmentation approaches				
			WOA	GWO	WOA-TH	GWO-TH	CCP
Church	3	Mean	3271.4427	3271.4383	3271.4427	3271.4445	**3276.8367**
		SD	1.24*E* – 02	9.93*E* – 03	**9.14 ** *E* **−** **13**	8.27*E* − 05	1.87*E* − 12
	5	Mean	3375.8721	3374.2197	3375.9118	3371.9813	**3381.9246**
		SD	2.65*E* + 00	6.74*E* + 00	2.62*E* + 00	9.65*E* + 00	**9.33*E*** − **13**
	8	Mean	3416.6815	3415.4415	3417.0876	3415.6558	**3423.5841**
		SD	2.40*E* + 00	1.74*E* + 00	1.78*E* + 00	3.51*E* + 00	**2.72*E*** − **02**

Train	3	Mean	2606.5951	2611.4987	**2611.5081**	2611.5081	2606.5951
		SD	2.26*E* − 02	2.53*E − *02	4.57*E − *12	4.57*E − *12	**9.52** *E * *** − *13**
	5	Mean	2740.6749	2740.5534	**2740.6800**	2740.6784	2736.2331
		SD	1.57*E − *02	1.16*E − *01	5.77*E − *03	6.18*E − *03	**2.32** *E * *** − *03**
	8	Mean	2794.6931	2793.9232	**2794.7170**	2794.2733	2790.4901
		SD	5.57*E − *02	1.19*E *+* *00	1.90*E − *02	5.32*E − *01	**1.12*E − *02**

Roman	3	Mean	2138.7893	2138.7837	2138.8057	2138.7992	**2142.3440**
		SD	2.52*E − *02	2.62*E − *02	0.00*E *+* *00	1.69*E − *02	**9.33** *E * *** − *13**
	5	Mean	2218.6635	2218.7936	2218.9622	2218.9687	**2221.3056**
		SD	2.53*E* + 00	7.93*E − *01	1.11*E − *02	2.35*E − *04	**9.25** *E * *** − *13**
	8	Mean	2249.3768	2248.6408	2249.6457	2249.6080	**2251.7798**
		SD	1.28*E* + 00	1.17*E* + 00	1.37*E − *01	2.00*E − *01	**3.77** *E * *** − *02**

Starfish	3	Mean	2779.9252	2779.9167	2779.9252	2779.9214	**2784.2272**
		SD	3.20*E − *12	6.64*E − *03	3.20*E − *12	5.91*E − *03	**1.40** *E * *** − *12**
	5	Mean	2912.8532	2912.7058	2912.8562	2912.8371	**2916.2730**
		SD	1.18*E − *02	1.66*E − *01	5.48*E − *03	4.62*E − *02	**9.34*E*-04**
	8	Mean	2972.2218	2971.3725	2972.3479	2972.2159	**2974.0989**
		SD	1.21*E* + 00	1.19*E* + 00	7.36*E − *03	1.99*E − *01	**2.23*E − *03**

Surfer II	3	Mean	7953.4167	7953.4063	7953.4167	7953.4135	**7953.4181**
		SD	5.07*E − *03	1.89*E − *02	4.82*E − *03	7.80*E − *03	**6.48** *E * *** − *12**
	5	Mean	8025.0703	8025.4959	8025.6049	8025.6025	**8025.6055**
		SD	7.73*E − *03	5.56*E − *02	7.11*E − *03	3.20*E − *03	**2.80** *E * *** − *12**
	8	Mean	8055.4215	8054.4135	8056.0617	8055.8463	**8056.0646**
		SD	8.27*E − *03	7.36*E − *01	2.05*E − *03	1.25*E − *01	**2.01** *E * *** − *03**
Cow	3	Mean	3858.2202	3858.2188	3858.2202	3858.2202	**3858.2202**
		SD	5.94*E − *12	1.03*E − *02	5.94*E − *12	5.94*E − *12	**1.85** *E * *** − *12**
	5	Mean	3965.2795	3965.1739	3965.2932	3964.6096	**3965.2932**
		SD	2.01*E − *02	9.72*E − *02	6.40*E − *12	2.93*E* + 00	**1.39** *E * *** − *12**
	8	Mean	4028.9410	4027.7892	4028.8474	4027.7210	**4028.9605**
		SD	1.83*E − *02	9.47*E − *01	6.11*E − *01	1.69*E* + 00	**1.50** *E * *** − *02**

Crocodile	3	Mean	3155.4359	3155.4337	3155.4359	3155.4359	**3155.4359**
		SD	9.14*E − *13	1.39*E − *02	9.14*E − *13	**9.14 ** *E * *** − *13**	9.33*E − *13
	5	Mean	3291.4340	3291.2945	3291.4366	3291.4287	**3291.4407**
		SD	1.02*E − *02	1.22*E − *01	5.38*E − *03	1.35*E − *02	**4.67*E − *13**
	8	Mean	3348.0384	3347.4528	3348.7453	3348.6504	**3348.7454**
		SD	2.79*E *+* *00	1.75*E *+* *00	**1.94 ** *E * *** − *03**	7.60*E − *02	3.27*E − *03

Elephants	3	Mean	1626.7183	1626.7192	1626.7205	1626.7196	**1626.7205**
		SD	3.53*E − *03	9.10*E − *03	1.37*E − *12	9.25*E − *03	**9.25** *E * *** − *13**
	5	Mean	1695.0482	1695.3208	1695.8664	1695.8231	**1695.8676**
		SD	3.99*E *+* *00	2.84*E *+* *00	**1.05 ** *E * **- ** * * **03**	8.42*E − *02	7.12*E − *02
	8	Mean	1727.1725	1726.5379	1728.2596	1728.1452	**1728.2676**
		SD	2.86*E* + 00	1.34*E* + 00	2.55*E − *02	1.35*E − *01	**1.20*E − *02**

## Data Availability

The two artificial datasets that were manually generated and often used in the existing literature are from the artificial datasets, available at https://www.isical.ac.in/content/databases (accessed June 2018). The eight real-life datasets are often used in the existing literature from the UCI Machine Learning Repository, available at http://archive.ics.uci.edu/ml/datasets.html (accessed June 2018). The eight tested images are from the Berkeley computer vision group, Berkeley segmentation dataset, and benchmark (BSDS300), available at https://www2.eecs.berkeley.edu/Research/Projects/CS/vision/grouping/segbench/(accessed October 2018).
